# Uniportal Video-Assisted Thoracoscopic Resection and Lobectomy for Infants With Pulmonary Sequestration: Case Series and Initial Experience

**DOI:** 10.3389/fped.2021.798342

**Published:** 2021-12-17

**Authors:** Jin-Xi Huang, Qiang Chen, Song-Ming Hong, Jun-Jie Hong, Hua Cao

**Affiliations:** ^1^Department of Cardiothoracic Surgery, Fujian Branch of Shanghai Children's Medical Center, Fuzhou, China; ^2^Fujian Children's Hospital, Fuzhou, China; ^3^Fujian Maternity and Child Health Hospital, Affiliated Hospital of Fujian Medical University, Fuzhou, China; ^4^Fujian Key Laboratory of Women and Children's Critical Diseases Research, Fujian Maternity and Child Health Hospital, Fuzhou, China

**Keywords:** video-assisted thoracoscopic surgery, infant, lobectomy, pulmonary sequestration, uniportal

## Abstract

**Background:** The present study aimed to evaluate the safety and feasibility of uniportal video-assisted thoracoscopic surgery (U-VATS) for infants with pulmonary sequestration (PS).

**Methods:** From January 2019 to July 2020, 19 infants with PS were admitted to a provincial hospital in the Fujian Province of China. A 1.5-cm utility port was created in the fifth intercostal space at the anterior axillary line. A rigid 30° 5-mm optic thoracoscope was used for vision, and two or three instruments were utilized through the port. Surgical options include standard lobectomy, wedge resection, and resection of the extralobar sequestration. Only one intercostal space was entered, and a chest tube was inserted through the same skin incision if necessary.

**Results:** The procedure was successful in all patients with an average operation duration of 58.3 ± 31.5 min. The length of post-operative hospital stay was 5.4 ± 1.5 days, and no post-operative deaths or serious complications were observed. The mean post-operative drainage volume was 164.6 ± 45.9 mL, and the mean post-operative thoracic tube indwelling duration was 5.5 ± 1.0 days. No intraoperative conversion, surgical mortality, or major complications were identified among the patients.

**Conclusion:** Our preliminary experience presented a series of U-VATS lobectomy, wedge resection, and resection of the PS for infants with satisfactory perioperative results.

## Introduction

Pulmonary sequestration (PS) is the second most common type of congenital dysplasia of the lungs and a non-functioning lung mass with an abnormal connection to tracheobronchial trees and an anomalous systemic artery supply (1, 2). The two subtypes of PS are intralobar sequestration (ILS) and extralobar sequestration (ELS). Patients with PS usually manifest upper airway syndrome, hemoptysis, or repeated lung infections (2). The infection rate in children with ILS was 71.17%, and that in children with ELS was 31.37% (3). The currently available data suggested that early (<6-months-old) surgery allows for ease of operative intervention, adequate recovery, and a reasonable time for compensatory lung growth while avoiding the potential infectious complications (4). Video-assisted thoracoscopic surgery (VATS) is a common and effective surgical method for PS, and lobectomy and lung-sparing resection are the two major operative approaches (3, 4).

Nearly a decade after the initial reports from Rocco et al. on the use of video-assisted thoracoscopic surgery (U-VATS) for intermediate therapeutic procedures in the form of non-anatomical wedge resection of nodules (5), the first lobectomy was performed by Gonzalez et al. (6), and Successive reports of anatomical segmentectomies (7), pneumonectomies (8), complex bronchial (9), and en bloc chest wall resections (10) followed. Shaqqura et al. (11) reported on U-VATS lobectomy in a 9-week-old patient, Chang SL reported three cases of one-lung ventilation using uniblocker bronchial blockers for infants in U-VATS (12), but no other study has yet described the U-VATS for infants with PS. The children's chest includes small cavum space and narrow intercostal space, making it challenging to perform U-VATS compared to other surgical procedures, such as multiportal VATS (13). Among clinical advantages, pain control after U-VATS has been reported to be superior to conventional, three-port access VATS (14). The survival and impact of the potentially limited immune response to trauma are areas that lack extensive studies, U-VATS lobectomy has been shown to reduce the humoral immune response to trauma (13). However, there was another answer to the question “why do a U-VATS lobectomy”: embrace the challenge is the only way to grow (15). The aim of this study is to evaluate the safety and feasibility of U-VATS for infants. The results are encouraging.

## Patients and Methods

The present study adhered to the tenets of the Declaration of Helsinki and was approved by the Ethics Committee of our hospital. Additionally, the written informed consent was obtained from the parents of the patients.

### Patients

The selection criteria were as follows: (1) Patients with PS, including ILS or ELS; (2) the ILS lesion was localized to only one pulmonary lobe; (3) all the patients were newly treated by surgery. The exclusion criteria were patients with other preoperative complications, such as congenital heart disease, immunocompromised state, restrictive or obstructive chest wall disease, and multiple lesions. From January 2019 to July 2020, two infants with complications (one with foregut cyst and another had coexistent left lower lobe ELS and left upper lung congenital cystic adenomatoid malformation) were excluded, and 19 infants with PS received U-VATS in our institution. Preoperative enhanced computed tomography (CT) was used to confirm the diagnosis, identify the lesions, and locate the systemic feeding artery. Routine examinations included a standard electrocardiogram, echocardiography, and blood tests. Subsequently, seven patients were found to be symptomatic, which included frequent respiratory infections and shortness of breath. Demographics, characteristics of PS (location, subtype, and origin of systemic artery), and perioperative data (operation time, blood loss, complications, duration of pleural drainage, and duration of post-operative hospital stay) were recorded for all patients.

### Surgical Technique

All patients were consecutively operated on by the same experienced thoracic surgical team. Herein, we used a uniportal approach as described and defined by Migliore et al. (16). All patients were operated under general anesthesia with selective one-lung ventilation using a single-lumen endotracheal tube with a bronchial blocker. The patients were positioned in the full lateral decubitus position, and the surgeon always stood at the ventral side of the patient. A 1.5-cm utility port was created in the fifth intercostal space (ICS) at the anterior axillary line. A wound protector (Disposable wound protectors, Changzhou Anker Medical Co., Ltd., Changzhou, China) was applied at the utility port. A rigid 30° 5-mm optic thoracoscope was used for vision, and two or three instruments, such as suction/irrigator device, electrocautery, endoscopic grasper or scissors, and endoscopic ultrasonic scalpel or open surgical instruments, were used through the utility port.

The majority of the dissections were performed using endoscopic hook electrocautery and a 5-mm endoscopic ultrasonic scalpel (Harmonic Scalpel; Ethicon Endo-Surgery Inc., Cincinnati, OH, USA). The systemic feeding artery was the first structure dissected out. It was divided using Hem-o-Lok clips (Sinolinks clips; Sinolinks Medical Innovation Inc., Jiangsu, China) at the beginning of the operation. The extent of parenchymal resection depended on the extent of PS. The surgical options include standard lobectomy, wedge resection, and resection of the ELS. Endo-GIA (Covidien, Mansfield, MA, USA) and Hem-o-Lok were used to carry out lobectomy. Because of the small incision, we use a 3-mm thoracoscope and a 1.2-mm forceps for operation when Endo-GIA was used. When the lesion is large, we cut the specimen into small pieces before extracting out. One chest tube was placed for pleural drainage after lobectomy or wedge resection, and no chest tube was placed after resection of the ELS. The chest tube was removed when there was no air leak, and the amount of daily drainage was <20 mL. Patients were discharged 1 day after removal of the chest tube if the follow-up chest X-ray did not show any signs of complications, such as pneumothorax and hydrothorax.

### Statistical Analysis

Continuous data are presented as mean ± standard deviation and range, and the categorical variables are presented as frequencies (%). Clinical parameters are shown in Table 1. SPSS (Windows version 19.0 IBM Co., Armonk, NY, USA) was used for all statistical analyses.

**Table 1:** Clinical data of infants undergoing U-VATS.

**Patient number**	**Age (months)**	**Gender**	**Weight (kg)**	**Type (ILS/ELS)**	**Location**	**Origin of systemic artery**	**Symptomatic**	**Operation ways**	**Operative time (min)**	**Blood loss (mL)**	**Drainage duration (days)**	**Drainage volume (mL)**	**Hospital stay (days)**
1	4	M	7.8	ILS	LLL	Thoracic aorta	Frequent respiratory infection	Wedge-shaped	60	20	6	220	7
2	4	F	10.1	ELS	RLL	Thoracic aorta	No		15	1			4
3	4	M	7.5	ILS	LLL	Thoracic aorta	No	Wedge-shaped	60	15	6	210	7
4	3	M	5.65	ELS	LLL	Thoracic aorta	Frequent respiratory infection		38	5			3
5	5	M	9.65	ILS	LLL	Thoracic aorta	Shortness of breath	Lobectomy	110	85	5	170	6
6	5	M	7.5	ILS	RLL	Thoracic aorta	Frequent respiratory infection	Lobectomy	120	80	4	120	5
7	6	F	6.9	ILS	LLL	Celiac trunk	No	Lobectomy	100	75	5	160	6
8	3	F	5.1	ELS	LLL	Superior mesenteric artery	No		40	10			4
9	4	M	6.9	ILS	RLL	Thoracic aorta	Frequent respiratory infection	Wedge-shaped	50	10	5	120	6
10	5	F	6.5	ILS	LLL	Thoracic aorta	No	Wedge-shaped	55	20	6	155	7
11	4	F	6.75	ELS	LLL	Abdominal aorta	No		20	1			4
12	6	M	9	ILS	LLL	Abdominal aorta	No	Wedge-shaped	60	20	6	200	7
13	3	M	5.5	ELS	RLL	Thoracic aorta	No		35	5			3
14	5	F	8	ILS	RLL	Thoracic aorta	Frequent respiratory infection	Wedge-shaped	65	25	7	220	8
15	5	M	7.5	ELS	LLL	Intercostal aorta	No		10	1			4
16	4	F	6.5	ILS	RLL	Thoracic aorta	Shortness of breath	Wedge-shaped	70	50	7	200	7
17	5	M	8.5	ILS	LLL	Thoracic aorta	No	Lobectomy	95	50	5	100	6
18	6	M	9.1	ILS	LLL	Abdominal aorta	No	Lobectomy	75	30	4	100	5
19	5	M	7.6	ELS	RLL	Abdominal aorta	No		30	2			4

*RLL, right lower lobe; LLL, left lower lobe*.

## Results

### Medical History and Diagnosis

A total of 19 infants (12 males and 7 females), aged 34.5 ± 0.9 (range: 3–6) months and weight 7.5 ± 1.4 (5.1–10.1) kg were recruited in this study. Most patients with PS had manifestations of lung infection, while some patients also presented dry cough and shortness of breath. PS was detected by prenatal ultrasonography in all patients. A total of 12 patients had ILS, and 7 had ELS. ILS was observed in the left lower lobe in 8 patients and the right lower lobe in 4 patients. Extralobar PS was detected near the left lower lobe in 4 patients and near the lower right lobe in 3 patients. The anomalous arterial blood supplies was via the thoracic aorta in 12 patients, abdominal aorta in 4, the celiac trunk in 1, intercostal aorta in 1, and superior mesenteric artery in 1 patient.

### Intraoperative Conditions and Post-operative Management

The surgery was smooth in all 19 patients. The operative time was 58.3 ± 31.5 (10–120) min, and the intraoperative blood loss volume was 26.6 ± 27.9 (1–85) mL. All 7 patients with ELS underwent resection of the diseased lung tissues. Among 12 patients with ILS, lobectomy was performed in five patients and wedge-shaped lung resection in seven patients. Systemic artery identification and ligation were completed in all cases. The mean post-operative drainage volume was 164.6 ± 45.9 mL, and the mean post-operative thoracic tube duration was 5.5 ± 1.0 days. No intraoperative conversion, surgical mortality, or major complications were observed among these patients. However, in some patients, respiratory symptoms, such as shortness of breath and repeated pneumonia, were noted before surgery, but none displayed any symptoms after surgery. The operative findings and post-operative outcomes are summarized in Table 1.

### Follow-Up

**Figure 1:**
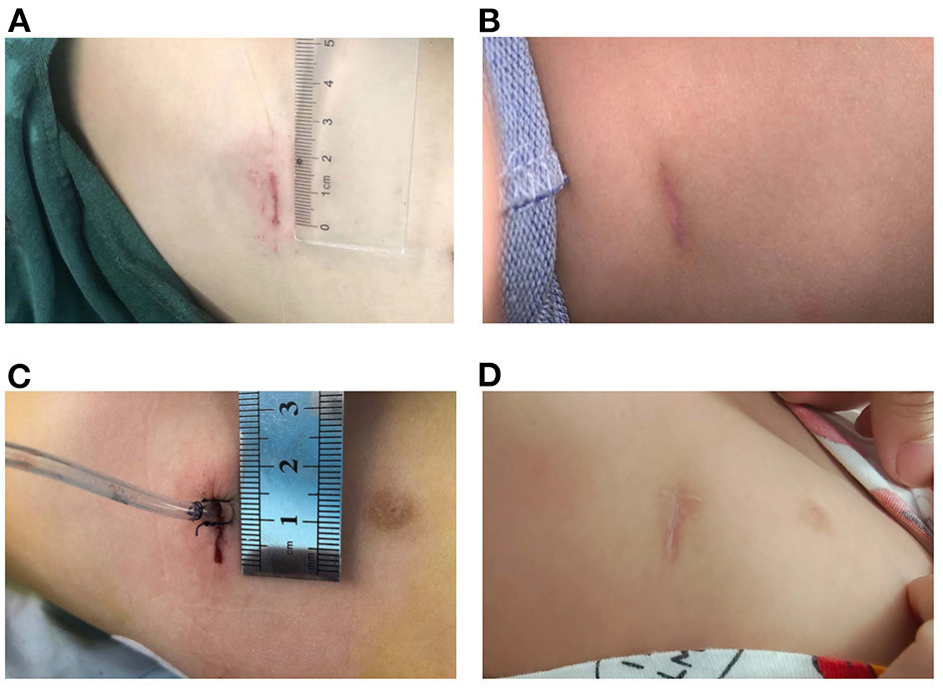
Incisions were minor and cosmetic **(A)** Incision without tube. **(B)** Incision without tube 3 months after surgery. **(C)** Incision with a chest tube. **(D)** Incision with a chest tube 3 months after surgery.

Follow-up and chest radiographs were performed 1-month post-surgery, and chest CT assessment was performed 1 year after surgery. All 19 patients were followed up for 1 year without loss. The symptoms had been either resolved totally or improved significantly in all symptomatic patients, and no complications were detected during the follow-up period. During a 2-year follow-up period after surgery, none of the patients developed symptoms. The incision in the chest was minor and cosmetic (Figure 1).

## Discussion

PS is an uncommon congenital anomaly due to the continuous advances in VATS. The approach has become the main surgical technique in treating PS (17). The surgical resection of the diseased lung is recommended for symptomatic patients and should also be performed for some asymptomatic patients to control and prevent recurrent pulmonary infection by blocking the inflammatory process and lowering the incidence of airway complications (1). The feasibility and safety of VATS lobectomy for PS have been investigated (18, 19).

For PS, U-VATS lobectomy has been applied to both ELS (20, 21) and ILS (22–24). However, these reported cases were all in adults and children after 1 year of age. Komori et al. examined long-term pulmonary function in 93 patients who underwent lobectomy between 2 days and 15 years of life (25). Alveolar growth (as opposed to emphysematous change) as measured by radionucleotide imaging was lower in patients who had undergone lobectomy after 1 year of age, suggesting an improvement in long-term pulmonary function in those who had undergone lobectomy before 1 year of age. Other than the case report from Shaqqura et al. (11), no studies have yet been reported about U-VATS in infants.

**Figure 2:**
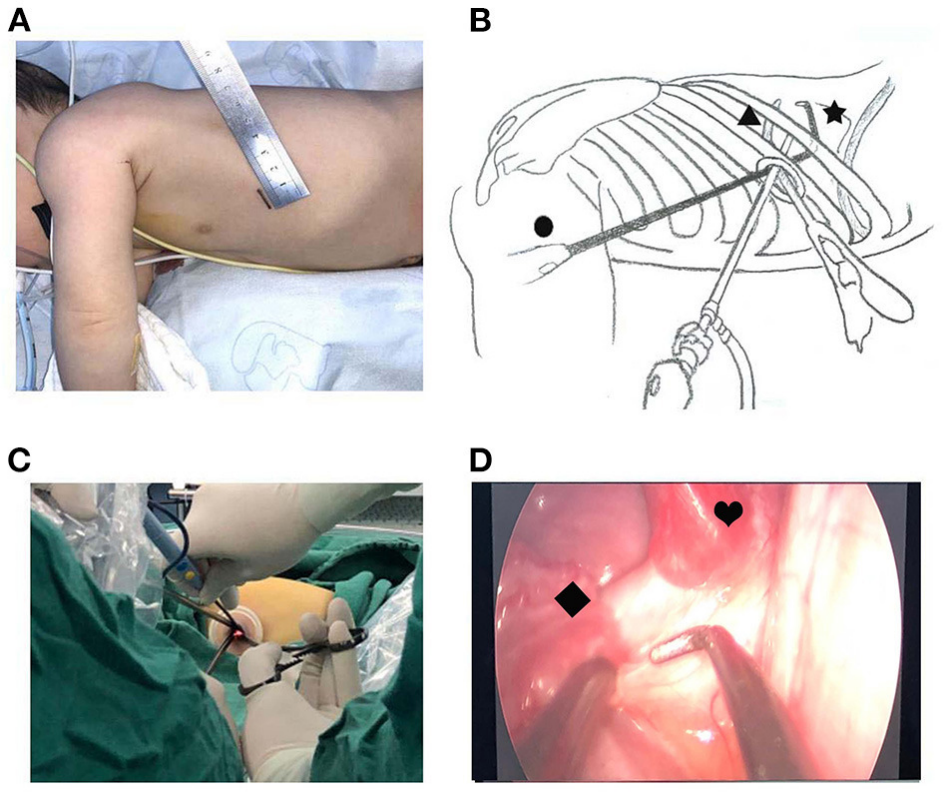
Uniportal thoracoscopic resection of pulmonary sequestration. **(A)** Location and length of the U-VATS incision. **(B)** •, thoracoscope; ▴, angulated forceps; ✶, plasticity electrocoagulation hook. **(C)** Operation photos. **(D)** Thoracoscopic image. ♥, PS; ♦, systemic feeding artery.

There is no consensus on the location and length of the U-VATS incision in infants. For adults, a 3 to 5-cm utility port was created in the fourth or fifth ICS at the anterior axillary line. In our opinion, a 4-cm incision is extremely traumatic for an infant. Hence, we recommend a 1.5-cm incision in the fifth ICS at the anterior axillary line for U-VATS. The pulmonary ligament and the aberrant systemic artery could be approached easily. Some characteristics of the infant's chest were small cavum space and narrow intercostal space. The distance between the surgical area and the chest surface was short, which resulted in a limited angle of instrument activities. Angulated and narrow-shaft double-hinged instruments have become a part of the essential armamentarium for the U-VATS, while articulating instruments bring the operative fulcrum inside the chest. The customized biarticular surgical instruments with a rod diameter of 4 mm were used in the current U-VATS, and the electrocoagulation hook was able to change the angle at will with a rod diameter of 2 mm. All the instruments made it advantageous to operate from multiple angles and in various regions (Figure 2).

**Figure 3:**
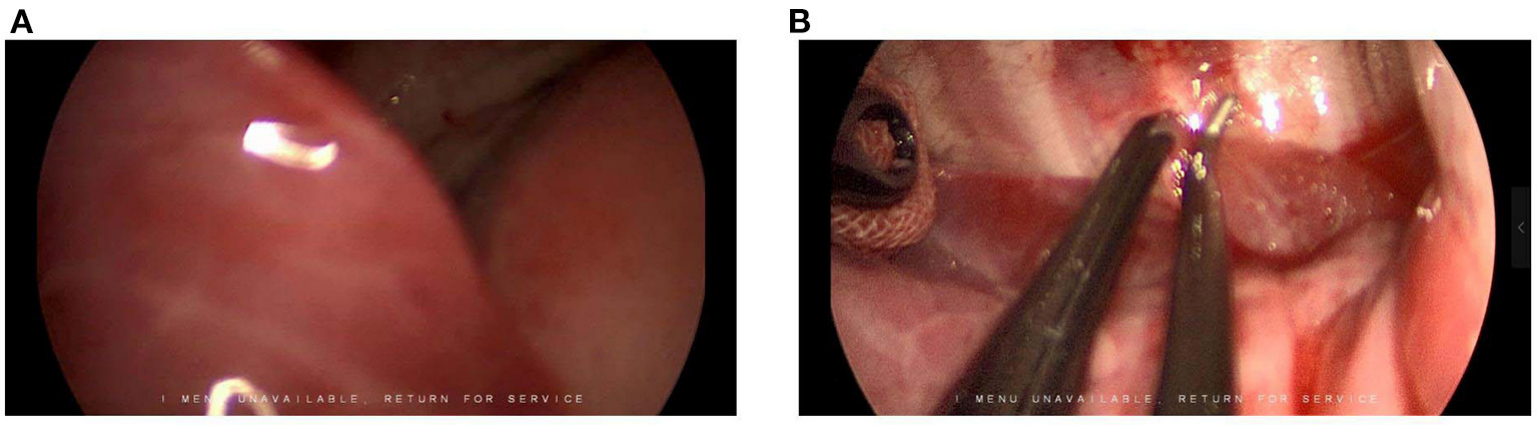
**(A,B**) Expose the surgical area.

The U-VATS for infants require close cooperation with the anesthesiologist and the camera-holding assistant. Maintaining the collapse of the lung on the operated side is also crucial. However, due to the small volume of the infant's chest cavity, the space with the collapsed lung was not sufficient to expose the surgical area. We also observed that the occlusion of the main bronchus, combined with the use of surgical instruments to remove the lobes blocking the view, produces satisfactory results. Notably, the lung tissue of the infants is vulnerable to being clipped by surgical instruments, and hence an angulated forceps holding a small piece of gauze was used to brush away the lobe from the surgical area (Figure 3).

The application of the U-VATS technique in thoracic surgery is a milestone innovation. However, there is often a long learning curve for the conversion from the conventional multiportal VATS to U-VATS. Our surgeons have had several years of experience in adult U-VATS; thus, we aimed to evaluate the safety and feasibility of U-VATS for infants in this study. Despite the success of 19 cases described above, the present study has many limitations; it was a single-center, small sample study, a retrospective analysis, and lacked a control group. Therefore, in the future, additional cases and complex U-VATS procedures, such as segmentectomy, are required to confirm the current findings using prospective, comparative studies.

## Conclusion

Although U-VATS is challenging, the surgical approach is safe and feasible for infants with PS. Herein, we presented a series of U-VATS lobectomy, wedge resection, and resection of the PS for infants with satisfactory perioperative results.

## Data Availability Statement

The original contributions presented in the study are included in the article/supplementary material, further inquiries can be directed to the corresponding author/s.

## Ethics Statement

The studies involving human participants were reviewed and approved by the Ethics Committee of Fujian Children's Hospital. Written informed consent to participate in this study was provided by the participants' legal guardian/next of kin.

## Author Contributions

J-XH participated in clinical practice, contributed to the collection and analysis of data, and drafting and revising the manuscript. J-JH carried out data collection. QC participated in clinical practice. S-MH helped in the study design and drafting of the manuscript. HC carried out patient recruitment and clinical practice, contributed to the conception and design of the study, and drafting and revising the manuscript. All authors read and approved the final manuscript.

## Conflict of Interest

The authors declare that the research was conducted in the absence of any commercial or financial relationships that could be construed as a potential conflict of interest.

## Publisher's Note

All claims expressed in this article are solely those of the authors and do not necessarily represent those of their affiliated organizations, or those of the publisher, the editors and the reviewers. Any product that may be evaluated in this article, or claim that may be made by its manufacturer, is not guaranteed or endorsed by the publisher.
